# Adolescents’ knowledge of and attitudes towards eating disorders

**DOI:** 10.1192/j.eurpsy.2022.1487

**Published:** 2022-09-01

**Authors:** A. Heneghan, M. Livanou

**Affiliations:** 1 Kingston University London, Department Of Psychology, Kingston upon Thames, United Kingdom; 2 Kingston University, Psychology, London, United Kingdom

**Keywords:** attitudes, eating disorder, knowledge, Adolescents

## Abstract

**Introduction:**

Eating disorders (EDs) constitute serious mental illnesses with high morbidity, lifetime mortality and associated stigma due to the label of mental illness. The sparse research assessing adolescents’ knowledge of and attitudes towards EDs highlights their low understanding of these conditions.

**Objectives:**

The proposed study aims to bridge this gap by investigating adolescents’ knowledge of and attitudes towards EDs as this will inform young people’s engagement with ED services.

**Methods:**

Participants aged 12-18 will be randomly assigned a vignette depicting either a male or female 15-year-old displaying symptoms of anorexia nervosa (AN) or binge eating disorder (BED). They will be asked to select what they believe the condition described in the vignette is from a pre-determined list. They will then be informed of the correct diagnosis before completing a series of scales designed to assess their attitudes towards EDs. Participants’ own potentially disordered eating behaviours will be assessed using the ED risk composite (EDRC) subscale from the EDI-3.

**Results:**

It is expected that BED will be less likely to be correctly identified compared to AN, eliciting more stigma and male vignette subjects will be seen more negatively than female vignette subjects. Also, it is expected that participants with higher EDRC scores will have more knowledge of and less negative attitudes towards EDs than those with lower EDRC scores.

**Conclusions:**

This study will highlight the need for education around EDs targeted at adolescents to increase their knowledge and awareness, providing them with factual information ought to reduce stigma and negative attitudes and beliefs about EDs.

**Disclosure:**

No significant relationships.

